# Identification of Residues of SARS-CoV nsp1 That Differentially Affect Inhibition of Gene Expression and Antiviral Signaling

**DOI:** 10.1371/journal.pone.0062416

**Published:** 2013-04-29

**Authors:** Andrew R. Jauregui, Dhruti Savalia, Virginia K. Lowry, Cara M. Farrell, Marc G. Wathelet

**Affiliations:** 1 Infectious Disease Program, Lovelace Respiratory Research Institute, Albuquerque, New Mexico, United States of America; 2 Department of Pathology, University of New Mexico, Albuquerque, New Mexico, United States of America; University of Tennessee Health Science Center, United States of America

## Abstract

An epidemic of Severe Acute Respiratory Syndrome (SARS) led to the identification of an associated coronavirus, SARS-CoV. This virus evades the host innate immune response in part through the expression of its non-structural protein (nsp) 1, which inhibits both host gene expression and virus- and interferon (IFN)-dependent signaling. Thus, nsp1 is a promising target for drugs, as inhibition of nsp1 would make SARS-CoV more susceptible to the host antiviral defenses. To gain a better understanding of nsp1 mode of action, we generated and analyzed 38 mutants of the SARS-CoV nsp1, targeting 62 solvent exposed residues out of the 180 amino acid protein. From this work, we identified six classes of mutants that abolished, attenuated or increased nsp1 inhibition of host gene expression and/or antiviral signaling. Each class of mutants clustered on SARS-CoV nsp1 surface and suggested nsp1 interacts with distinct host factors to exert its inhibitory activities. Identification of the nsp1 residues critical for its activities and the pathways involved in these activities should help in the design of drugs targeting nsp1. Significantly, several point mutants increased the inhibitory activity of nsp1, suggesting that coronaviruses could evolve a greater ability to evade the host response through mutations of such residues.

## Introduction

Vertebrate cells use an array of sensors to detect infection by viruses and other microorganisms; triggering of such sensors activates signaling cascades that lead to the expression of host defense genes necessary to fight off the infection [1]. Naturally, viruses evolved to encode not only proteins necessary for their replication, such as their polymerase and capsid protein, but also security proteins that counteract the host defenses [2]. As unimpeded induction and action of interferons (IFNs) in response to viral infection leads to a powerful antiviral state that strongly restricts replication of most viruses [3], viral IFN antagonists constitute a major class of security proteins [4], [5]. IFN antagonists can be further distinguished based on their ability to i) inhibit the virus-dependent signaling necessary for the production of IFNs and other cytokines; ii) inhibit the IFN-dependent signaling necessary for the induction of antiviral genes; or iii) inhibit the activity of antiviral proteins.

The Severe Acute Respiratory Syndrome (SARS) Coronavirus (CoV) is an enveloped, positive-stranded RNA viruses that can cause a severe respiratory disease [6]–[12]. Its genome consists of a ∼30 kb linear, non-segmented, capped, polycistronic, polyadenylated RNA molecule, the first two-third of which is directly translated into two large polyproteins. These two polypeptides are processed into 16 non-structural proteins (nsps), forming the replicase complex, which is active in the cytoplasm in close association with cellular membranes. Transcription and replication of the coronaviral genome leads to the expression of open reading frames located in the remaining third of the genome, which encode structural proteins such as S (spike), E (envelope), M (membrane) and NP (nucleocapsid protein), as well as other open reading frames (ORFs) whose functions remain to be fully characterized [6]–[9].

A number of putative IFN antagonists have been identified in the SARS-CoV genome through a variety of screens: nsp1, nsp3, nsp7, nsp15, ORF3a, ORF3b, ORF6, M and NP [13]–[24]. However, validation of their role as genuine IFN antagonists in the context of the live virus was obtained only for nsp1 so far [16], [25]. Two mechanisms were identified to account for the IFN antagonist activity of nsp1, general inhibition of host gene expression [13], [16], [25] and inhibition of antiviral signal transduction [16]. Specifically, nsp1 inhibits host gene expression by decreasing translation efficiency and by destabilizing mRNAs [13], [25], [26]. Additionally, nsp1 expression inhibits the three virus-dependent signaling pathways that lead to activation of the transcription factors ATF2/c-Jun, IRF3/IRF7, and NF-κB, preventing full induction of virus-inducible genes, such as IFNB [16]; moreover, nsp1 decreases the levels of STAT1 phosphorylation, inhibiting the induction of genes by IFN-α and IFN-γ [16].

The pleiotropic activities of nsp1 could be the result of multiple host factors interacting with nsp1 or of a single factor whose interaction with nsp1 eventually impacts distinct cellular pathways. In this study, we carried out a detailed mutational analysis of SARS-CoV nsp1 to distinguish between these possibilities. We determined that SARS-CoV nsp1 main functional activities, inhibition of host gene expression and inhibition of antiviral signaling, could be genetically separated. Mapping of residues involved in inhibitory functions on the surface of nsp1 suggests it interacts with distinct host factors to inhibit host gene expression and antiviral signaling.

## Materials and Methods

### Cell Culture and Viruses

293T cells (ATCC CRL-11268) are a SV40 large T antigen-expressing and highly transfectable derivative of 293 cells, which are derived from human embryonic kidney cells transformed with human adenovirus type 5, were grown at 37°C, 5% CO_2_, in Dulbecco’s Modified Eagle medium containing 10% Hyclone bovine growth serum, 50 U/mL penicillin and 50 µg/mL streptomycin. Sendai Virus (SeV, from SPAFAS) was used at 25 hemagglutinin units (HAU)/mL. Viruses and cell lines were manipulated according to BSL-2 guidelines under a protocol approved by the Institutional Biosafety Committee.

### SARS-CoV nsp1 Mutants and Reporter Plasmids

Mutations in SARS-CoV nsp1 were introduced by PCR. All nsp1 coding regions were cloned into pcDßAF_3_m1 downstream of a N-terminal triple flag-tag sequence, as described [16], and verified by sequencing; primer sequences and plasmid sequences are available upon request. The virus- and interferon (IFN)-responsive reporter construct ISREx3CAT contains three copies of the ISG15 gene IFN-Stimulated Response Element (ISRE) and the TATA box of the E1b gene to drive expression of the Chloramphenicol Acetyl Transferase (CAT) gene [27], [28]. Plasmids for expression of IRF3, STAT1α, enhanced green fluorescent protein (eGFP), luciferase (*P. pyralis*), and β-galactosidase (lacZ) were described previously [16], [28], [29]. All proteins were expressed from the Cytomegalovirus (CMV) enhancer.

### Cell Transfections and Reporter Assays

293T cells (2.0×10^6^ in 100 mm dishes) were transfected by calcium phosphate coprecipitation [27]. Transfections consisted of either ISREx3-CAT reporter or pcDßA-STAT1α or pcDßA-IRF3, combined with pcDβAF_3_nsp1-wildtype (wt) or pcDβAF_3_nsp1-mutant (m1–m39) plasmid, as well as the following plasmids reporters: pCMV-lacZ, pcDβA-luciferase, and pcDβA-eGFP. Transfection efficiency is routinely >95% as determined by *in situ* lacZ staining [27] or eGFP expression. Transfected cells were trypsinized and aliquoted for individual treatment conditions (control, hIFNα, SeV). Cell extracts were made with M-Per (Pierce). Standardized CAT, luciferase and β-galactosidase assays were performed, with appropriate dilutions to remain in the linear range for each assay [27]: all extracts for CAT activity were diluted 100-fold and activity is computed as percent conversion, where the counts of mono-acetylated-[^14^C]-chloramphenicol is divided by total (mono-acetylated+un-acetylated)-[^14^C]-chloramphenicol counts (di-acetylated-[^14^C]-chloramphenicol is not formed when the assay is carried out in the linear range); luciferase activity is expressed as relative light units (RLU); all extracts for β-galactosidase activity were diluted 10-fold and activity is expressed in absorbance units at 405 nm that results from the release of ortho-nitrophenol from ortho-nitrophenyl-β-galactoside. Statistical significance was determined using a two-tail t-Test assuming unequal variances. All experiments replicated a minimum of three times.

### Immunoblot Analysis

Cell extracts were analyzed by sodium dodecyl sulfate (SDS)-polyacrylamide gel electrophoresis (PAGE) and immunoblotting [30] with the following commercial primary antibodies: anti-STAT1 (sc-592, Santa Cruz Biotech.); anti-phospho-Tyr701-STAT1 (#9171L, Cell Signaling) to detect levels of total and phosphorylated STAT1; and anti-FLAG-M2 (Sigma) to detect FLAG-tagged nsp1 proteins. IRF3 was detected following deoxycholate (DOC)-PAGE as described [29]; monoclonal SL-12 (BD Pharmingen) detects both IRF3 monomers and dimers. Primary antibodies were detected by immunoglobulin-horseradish peroxidase conjugated secondary antibody (Promega) and visualized using the PerkinElmer Life Sciences chemiluminescence detection system. Bradford Assay was used to determine protein concentration of extracts, and ten micrograms of total protein per sample was loaded onto gels. Quantitation of immunoblots was done using ImageJ [31]. To calculate relative STAT1 phosphorylation, the quantity of phosphorylated STAT1 was divided by the quantity of total STAT1 for each time point. To calculate the relative IRF3 dimerization, the quantity of IRF3 dimer was divided by the quantity of IRF3 monomer plus the dimer for each time point. All experiments and measurements are replicated a minimum of three times.

## Results

### SARS-CoV nsp1 Likely Interacts with Multiple Host Factors

SARS-CoV nsp1-wt exhibits multiple inhibitory effects on host functions. In addition to inhibiting host gene expression and virus- and IFN-dependent signaling, SARS-CoV nsp1 also inhibits cell cycling and decreases cell death in transfected cells [16]. We hypothesized that these pleiotropic activities could be the result of multiple interactions between nsp1 and host proteins. If true, the prediction would be that individual residues in nsp1, which if mutated, would differentially affect the ability of nsp1 to inhibit host activities; if untrue no such mutations would be identified.

**Figure 1:**
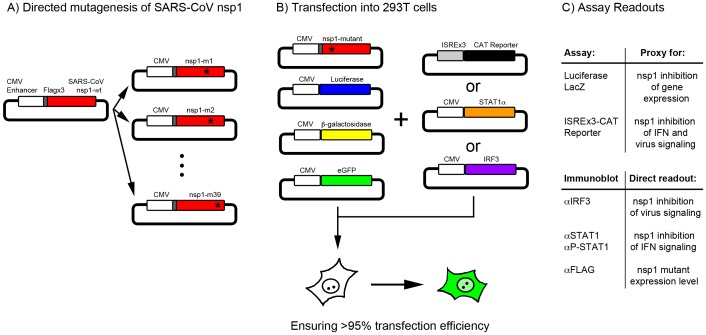
Graphical representation of experimental design. (A) SARS-CoV nsp1 was cloned into a plasmid driven by the CMV enhancer with a N-terminal triple FLAG tag. Surface residues that could be important for function were identified and mutated. (B) SARS-CoV nsp1-wt and mutants were cotransfected into 293T cells with reporter plasmids. Plasmid expressing eGFP was used to visually inspect for high transfection efficiency. (C) Standard assays were performed: luciferase and β-galactosidase assays were used as a proxy to measure level of nsp1 inhibition of gene expression; CAT gene under the control of three ISRE copies was used as a proxy for nsp1 inhibition of interferon- and virus-dependent signaling; immunoblots were run to directly measure nsp1 inhibition of STAT1 phosphorylation and IRF3 dimerization, and nsp1 levels.

To distinguish between these possible modes of action, we carried out a mutational analysis of SARS-CoV nsp1. Before the structure of SARS-CoV nsp1 was determined, we initially selected a few amino acids that were predicted to be solvent exposed (mutants m1–m8); after the NMR structure of SARS-CoV nsp1 was reported [32], we selected additional solvent exposed residues for replacement (mutants m9–m39). The nature of the selected residues was changed, e.g., positively charged to negatively charged, so that if the amino acid was involved in an interaction with a host factor, that interaction would likely be disrupted. This abrogation of nsp1 to host-protein interactions would be reflected in the attenuation of standard assays measuring the level of nsp1 inhibition of host functions. The nsp1 mutants were generated by PCR, cloned into plasmids (Figure 1A), and were co-transfected into 293T cells along with plasmids expressing luciferase, β-galactosidase, eGFP, and a reporter plasmid expressing the CAT gene under the control of a virus- and IFN-inducible ISRE, ISREx3-CAT (Figure 1B). Standardized luciferase and β-galactosidase assays were used as a proxy for an nsp1 mutant’s ability to inhibit host gene expression; inhibition of luciferase activity by nsp1-wt is mild (∼40%) while inhibition of β-galactosidase is strong (∼90%) [16]; other investigators used different reporters as proxy for nsp1 effects on host gene expression [13], [25], [33], [34]. Expression of the ISREx3CAT reporter is stimulated by both IFN treatment and SeV infection and coexpression of SARS-CoV nsp1-wt results in an inhibition of that stimulation, which we previously showed was due to an inhibitory effect on signaling and not on expression of the CAT gene [16]. Thus, CAT expression is used here as a proxy for an nsp1 mutant’s ability to inhibit virus- and IFN-dependent signaling. Immunoblots were run and quantitated to measure the expression levels of all nsp1 mutants in these experiments (Figure 1C). Direct measurement of virus- and IFN-dependent signaling was also carried out (see below).

**Figure 2:**
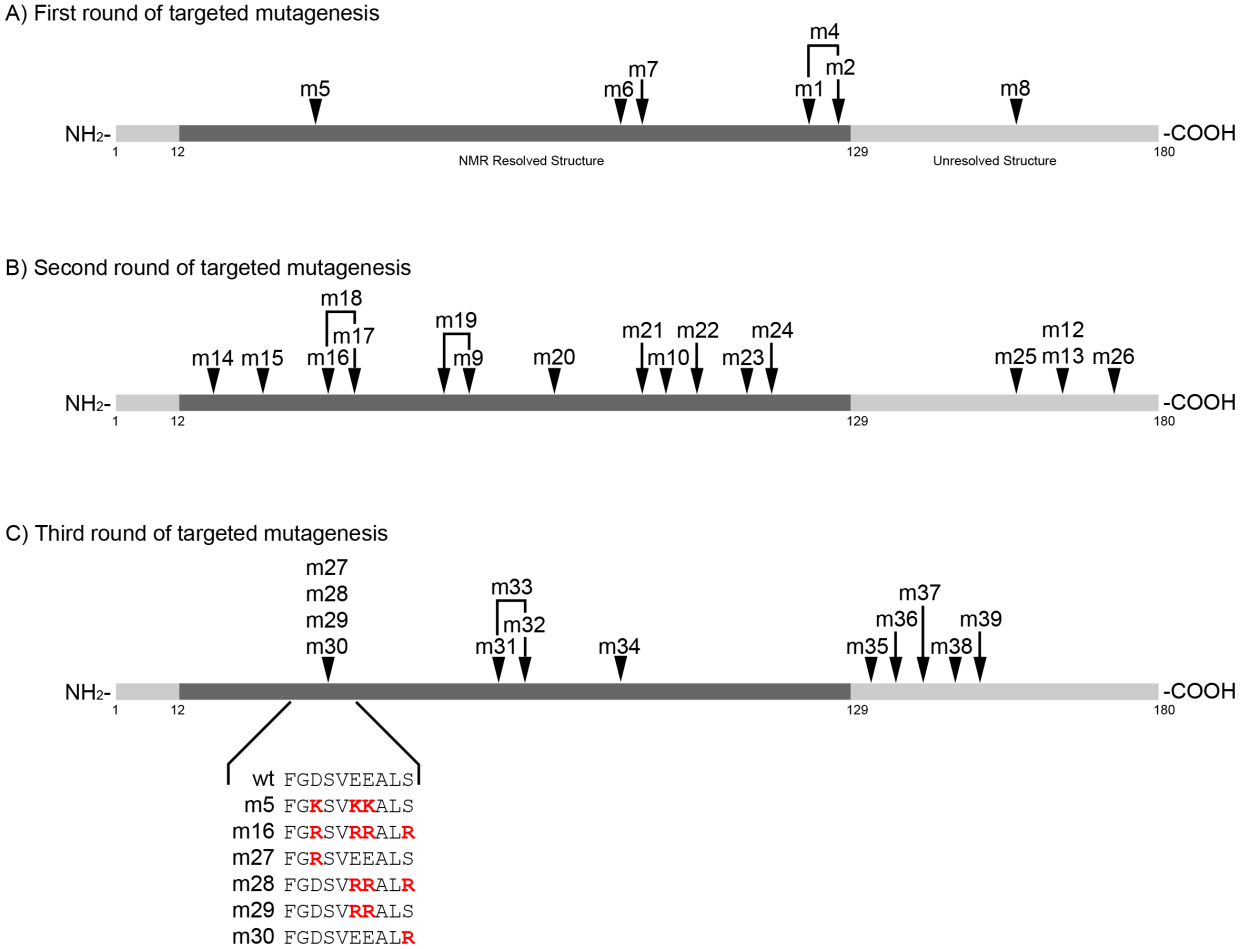
Map of SARS-CoV nsp1 mutants generated. SARS-CoV nsp1 is a small 180-residue protein whose structure has been solved by NMR for residues 13–129. We hypothesized that surface residues would be important for interaction between nsp1 and host target protein(s) that would mediate the inhibitory effects of nsp1. Three rounds of mutagenesis targeting surface residues were carried out in this study (A–C). Mutant nsp1-m5 was identified to potentially mediate nsp1 inhibition of host IFN- and virus-dependent signaling (A) but not inhibition of host gene expression and was explored further. Mutant nsp1-m16 was generated to attempt to further resolve the nsp1-m5 loss of function, and mutants -m27 through -m30 (C) were generated to refine important residues identified in nsp1-m16 (B).

**Figure 3:**
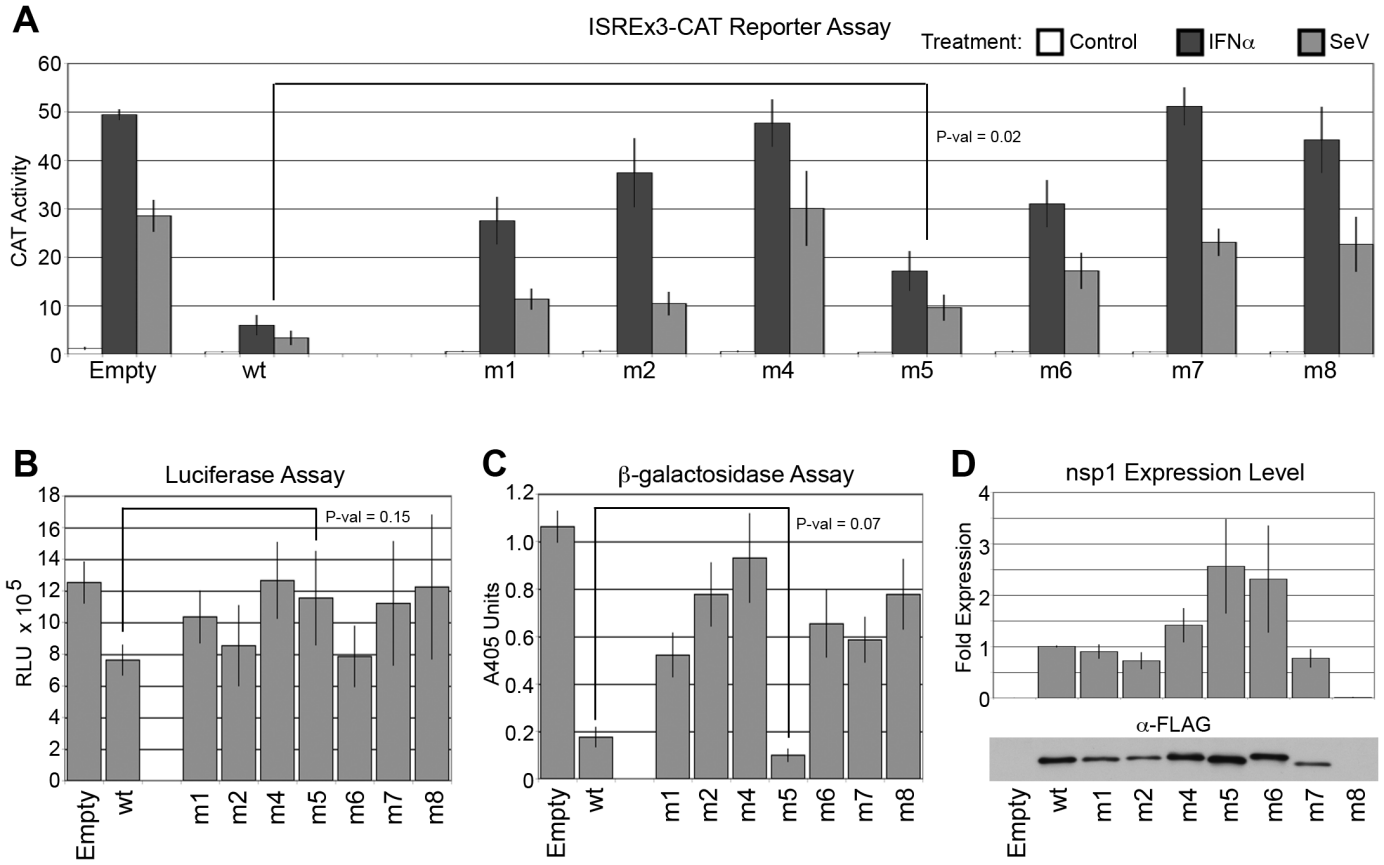
First round of mutagenesis reveals nsp1-m5 mutant with partial loss of inhibition of host signaling. SARS-CoV mutants nsp1-m1 through nsp1-m8 were tested for (A) inhibition of host IFN- and virus-dependent signaling using the ISREx3-CAT reporter, followed by inhibition of gene expression using (B) luciferase and (C) β-galactosidase assays. CAT activity values correspond to percent chloramphenicol acetylation using cell extracts diluted 100-fold, luciferase activity is determined in straight extracts and is expressed in RLU and β-galactosidase activity corresponds to released ortho-nitrophenol absorption at 405 nm using extracts diluted 10-fold. Immunoblots of nsp1 mutants are quantitated in (D). Error bars are ± standard error; P-values are result of a t-Test. Mutant nsp1-m5 exhibited attenuated inhibition of host IFN- and virus-dependent signaling (A) while maintaining wildtype inhibition of β-galactosidase (C); P-values for nsp1-m5 are indicated in figure, significance for other mutants is listed in Table 1.

Eight nsp1 variants were generated (nsp1-m1 through nsp1-m8) as an initial test of our hypothesis (Figure 2A). As expected, variants were discovered that exhibited attenuated (nsp1-m1) or almost complete loss of inhibition (nsp1-m4, -m7 and -m8) of both signaling (Figure 3A) and gene expression (Figure 3B,C). Expression of all nsp1 mutants was compared to that of nsp1-wt (Figure 3D); some variants, such as nsp1-m8, were consistently expressed at such low levels in transfected cells that we removed them from further consideration in our measurements (Table 1). Of particular interest was nsp1-m5, which exhibited a statistically significant attenuation of inhibition of antiviral signaling while maintaining a strong inhibition of β-galactosidase expression, similar to that obtained with nsp1-wt. Variant nsp1-m5 phenotype of partial inhibition of host signaling with strong inhibition of β-galactosidase expression is consistent with our hypothesis that nsp1 exerts its pleiotropic inhibitory effects by interacting with more than one host protein.

**Table 1:** Raw and normalized inhibitory activities for all nsp1 mutants.

			Inhibition									Normalized Inhibition			
			Gene expression				Signaling				nsp1 Fold	Gene expression		Signaling	
nsp1	Residue Mutation	Group	LacZ		Lucif		IFNα		Virus		Expression	LacZ	Lucif	IFNα	Virus
empty	–	–	0		0		0		0		–	–	–	–	–
wt	–	–	100	*	100	*	100	*	100	*	1.00	100	100	100	100
m1	**R124S, K125E**	**i**	34	*#	74	*#	21	*#	29	*#	0.90	37	82	24	32
m2	**N128S, K129E**	**i**	23	#	89		16	#	32	*#	0.72	31	124	22	44
m9	**K58E**	**i**	22	*#	78		17	#	21	*#	0.87	25	89	19	24
m10	**R99E**	**i**	25	*#	139	*#	19	*#	16	#	0.82	30	169	23	20
m12	**K164E, H165E**	**i**	21	*#	69	#	14	#	15	#	0.33	64	207	43	46
m18	**D33R, E36R, E37R, E41R, R43E, E44R, K47E**	**i**	27	#	111	*	20	*#	17	#	0.66	41	169	30	26
m21	**E91R, M92A, D93H, I95A, Q96H**	**i**	33	*#	111	*	15	#	19	#	0.57	58	194	26	34
m22	**S100R, G101R**	**i**	33	*#	205	*#	30	*#	36	*	1.05	31	195	29	35
m37	**K141D, S142Q**	**i**	110	*	158	*#	93	*	112	*	2.31	48	69	40	48
m13	**K164A, H165A**	**i**	21	*#	61	#	14	#	15	#	0.44	46	139	31	34
m35	**H134D, S135Q**	**i**	18	#	93	*	20	*#	26	*	0.97	18	96	21	27
m4	**R124S, K125D, N128S, K128D**	**ii**	19	#	60	#	12	#	11	#	1.41	13	43	9	8
m6	**H81E, H83E, K84D**	**ii**	27	*#	97	*	19	*#	19	*#	2.31	12	42	8	8
m8	**E155K, D156K, E158K**	**ii**	–	#	–	#	–	#	–	#	–	–	–	–	–
m25	**E155R, D156R, E158R**	**ii**	–	#	–	#	–	#	–	#	–	–	–	–	–
m26	**R171D, E172N, R175N, E176H**	**ii**	–	#	–	#	–	#	–	#	–	–	–	–	–
m17	**E41R, R43E, E44R, K47E**	**ii**	84	*	77		32	*#	36	*#	2.32	36	33	14	16
m7	**E91K, D93K**	**ii**	30	*#	68	#	12	#	14	#	0.77	39	88	15	19
m5	**D33K, E36K, E37K**	**iii**	177	*	66	#	35	*#	35	*#	2.56	69	26	13	14
m16	**D33R, E36R, E37R, S40R**	**iii**	58	*	61		13	#	17	#	0.92	63	67	14	19
m23	**V111D, G112D, T114A**	**iv**	80	*	80		324	*	400	*	1.28	63	63	254	314
m24	**A117E, Y118F, N120E**	**iv**	137	*	93	*	181	*	242	*	1.28	107	73	142	190
m32	**E65R**	**iv**	63	*	117	*	77	*	169	*	0.63	101	186	122	269
m14	**Q15R, S17H**	**v**	229	*#	373	*#	1838	*#	1767	*#	1.34	171	278	1373	1320
m19	**E55R, E57R, K58E, G59R**	**v**	138	*	114	*	113	*	109	*	0.30	455	376	373	361
m27	**D33R**	**v**	250	*#	547	*#	2065	*#	1770	*#	0.70	358	784	2960	2538
m28	**E36R, E37R, S40R**	**v**	74	*	94		127	*	176	*	0.53	139	177	239	331
m29	**E36R, E37R**	**v**	129	*	202	*#	58	*	103	*	0.36	356	556	160	285
m30	**S40R**	**v**	125	*	246	*#	868	*#	933	*#	0.58	215	422	1491	1603
m38	**D147R, E148R**	**v**	141	*	174	*#	726	*#	856	*#	1.07	131	162	676	796
m39	**T151Q, D152R**	**v**	171	*	202	*#	375	*#	631	*#	1.66	103	122	225	380
m31	**Q63R**	**v**	73	*	99		34	*	68	*	0.35	208	284	97	194
m33	**Q63R, E65R**	**v**	98	*	167	*#	30	*#	44	*	0.48	203	345	61	91
m15	**L27D, V28D**	**vi**	138	*	94	*	219	*	275	*	2.03	68	46	108	136
m20	**R73E, D75R, L77A, S78E, N80G**	**vi**	154	*	158	*#	108	*	132	*	1.13	136	140	96	117
m34	**K84E**	**vi**	88	*	104	*	76	*	153	*	1.67	53	62	46	92
m36	**D139R**	**vi**	74	*	81		58	*	58	*	0.72	102	112	81	80

The inhibitory functions for all nsp1 mutants are shown in this table expressed as averaged percent compared to nsp1-wt (set to 100 percent). Percent inhibitions were also normalized to expression levels. No values are reported for mutants nsp1-m8, -m25, and -m26 as they had no significant inhibitory activities and were expressed at a level too low to be measured. After expression normalization, many nsp1 mutants exhibited increased inhibitory abilities compared to nsp1-wt; for example, nsp1-m19 exhibits nearly four-fold more inhibitory functions over nsp1-wt. Even after normalization, nsp1-m16 continues to show a loss of inhibition of host IFN- and virus-dependent signaling while maintaining strong inhibition of host gene expression. (*indicates significant as compared to empty vector; ^#^indicates significant as compared to nsp1-wt; significant is defined as a t-Test P-value ≤ 0.05).

### Distinct Inhibitory Activities of SARS-CoV nsp1 can be Genetically Separated

We carried out a second round of nsp1 mutagenesis (nsp1-m9 through nsp1-m26, Figure 2B) to identify additional residues that may play a role in nsp1 activities, with particular attention to the region surrounding the residues mutated in nsp1-m5 (D33K, E36K, E37K) that appears to be involved in nsp1 inhibition of antiviral signaling.

**Figure 4:**
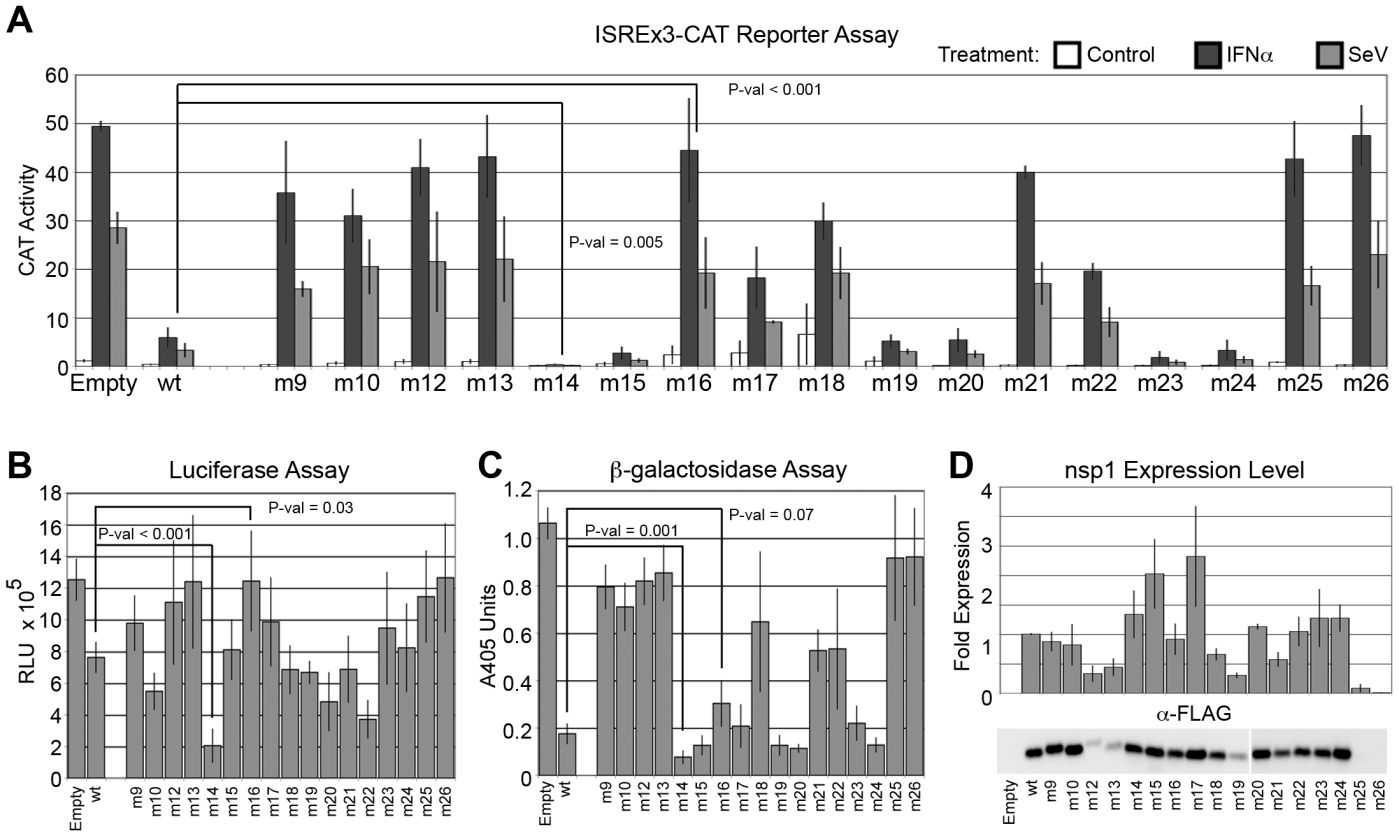
Second round of mutagenesis reveals nsp1-m16 mutant with complete loss of inhibition of host signaling. SARS-CoV nsp1-m9 through nsp1-m26 mutants were tested for (A) inhibition of host IFN- and virus-dependent signaling using the ISREx3-CAT reporter, followed by inhibition of host gene expression using (B) luciferase and (C) β-galactosidase assays. CAT activity values correspond to percent chloramphenicol acetylation using cell extracts diluted 100-fold, luciferase activity is determined in straight extracts and is expressed in RLU and β-galactosidase activity corresponds to released ortho-nitrophenol absorption at 405 nm using extracts diluted 10-fold. Immunoblots of nsp1 mutants are quantitated in (D). Mutant nsp1-m16 had completely lost its ability to inhibit host IFN- and virus-dependent signaling (A) while retaining wildtype levels of inhibition of β-galactosidase expression (C). Mutant nsp1-m14 consistently exhibited stronger inhibitory effects than nsp1-wt in all assays (A,B,C). Error bars are ± standard error; P-values are result of a t-Test. P-values for nsp1-m14 and -m16 are indicated in figure, significance for other mutants is listed in Table 1.

Analysis of the second round of mutants identified some noteworthy phenotypes (Figure 4). Mutants nsp1-m14, -m15, -m23 inhibited the virus- and IFN-inducible CAT reporter more strongly than nsp1-wt, with nsp1-m14 essentially abolishing any induction of the CAT reporter and more strongly inhibiting host gene expression than nsp1-wt as determined by both the luciferase and β-galactosidase assays. Variants nsp1-m16 (D33R, E36R, E37R, S40R) and nsp1-m17 (E41R, R43E, E44R, K47E) were selected for their immediate proximity to the residues mutated in nsp1-m5 to attempt to further separate the inhibition of antiviral signaling from the inhibition of host gene expression. Mutants nsp1-m17 and -m18 (incorporating both m16 and m17 mutations), were not significantly different from nsp1-m5 in their ability to inhibit the CAT reporter stimulation by IFN or SeV. By contrast, nsp1-m16 exhibited a complete loss of inhibition of antiviral signaling (Figure 4A), while maintaining strong inhibition of β-galactosidase expression (Figure 4C). In addition, mutations introduced in the intrinsically disordered C-terminal segment (amino acids 128–180) of nsp1, expressed relatively poorly (mutants nsp1-m12 and -m13) or barely at all (mutants nsp1-m25 and -m26, Figure 4D). Mutant nsp1-m25 targeted the same residues as nsp1-m8, but instead of replacing the acidic residues with lysines, arginine residues were substituted to test the possibility that the lack of nsp1-m8 expression was due to ubiquitination of those lysines; rather, our results suggest that the charge change was sufficient to interfere with expression of nsp1. Variants nsp1-m25 and -m26 were consistently expressed at such very low levels in transfected cells, displaying no inhibitory activities, that we removed them from further consideration in our measurements (Table 1).

Thus, we found that the disordered C-terminal tail, including a putative α-helix targeted by the m26 mutation, is important for proper expression of nsp1. Importantly, mutations were identified that increased the inhibitory effects of nsp1 on a virus- and IFN-inducible reporter, suggesting a potential for the evolution of increased evasion from the host response for SARS-CoV. With the generation of nsp1-m16, which further attenuated the nsp1-m5 phenotype, we confirm the hypothesis that the different inhibitory properties of nsp1 can be genetically separated.

**Figure 5:**
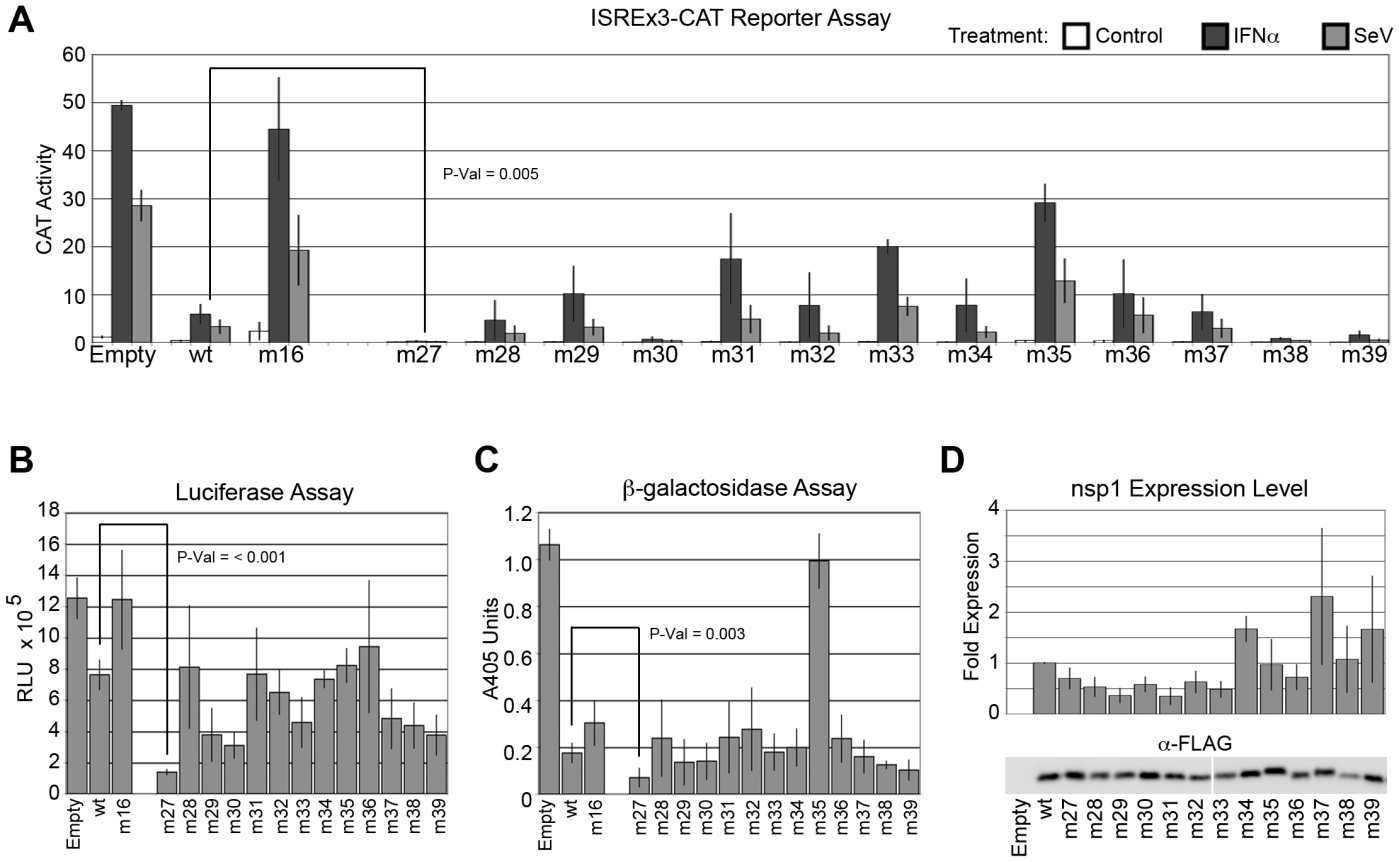
Third round of mutagenesis refines map of nsp1 residues important for inhibition of host signaling or gene expression. SARS-CoV nsp1-m27 through nps1-m39 mutants were tested for (A) inhibition of host IFN- and virus-dependent signaling using the ISREx3-CAT reporter, followed by inhibition of host gene expression using (B) luciferase and (C) β-galactosidase assays. CAT activity values correspond to percent chloramphenicol acetylation using cell extracts diluted 100-fold, luciferase activity is determined in straight extracts and is expressed in RLU and β-galactosidase activity corresponds to released ortho-nitrophenol absorption at 405 nm using extracts diluted 10-fold. Mutants nsp1-m27 through nsp1-m30 were derived from nsp1-m16 to define minimal residues important for inhibition of host signaling. Immunoblots of nsp1 mutants are quantitated in (D). Error bars are ± standard error; significance for all mutants is listed in Table 1.

A third round of mutations was carried out to further explore the surface of nsp1 and complete the map of functional residues. Mutants -m27 through -m30 were generated in an attempt to tease out residues accounting for the separation of inhibition of signaling and gene expression activities of nsp1-m16; mutants -m31 through -m34 targeted residues located on the surface of nsp1, midway between the functional domains emerging from the analysis of the first two rounds of mutations; and mutants -m35 through -m39 affected residues in the intrinsically disordered C-terminus of nsp1. Intriguingly, mutants -m27 through -m30 had a very different phenotype than nsp1-m5 or -m16 from which they were derived, with nsp1-m27 and -m30 inhibiting the IFN- and virus-inducible CAT reporter and both β-galactosidase and luciferase activities more strongly than nsp1-wt (Figure 5). The remaining mutants, nsp1-m31 through nsp1-m39, displayed no phenotype distinct from any of those already described in this study.

With the third round of nsp1 mutagenesis, we completed a map of functional residues important for the inhibition of gene expression and antiviral signaling. We were able to isolate a putative, minimal set of residues involved solely in the inhibition of host IFN- and virus-dependent signaling, but not in the inhibition of host gene expression. No mutations were identified that resulted in a loss of the inhibition of gene expression without also resulting in a loss of the inhibition of antiviral signaling and additional mutations were identified that increased the inhibitory effects of nsp1 on a virus- and IFN-inducible reporter.

### Attenuated nsp1 Mutants have Lost the Ability to Inhibit Antiviral Signaling

We next investigated the ability of a representative set of SARS-CoV nsp1 mutants to directly inhibit antiviral signaling by assessing STAT1 phosphorylation and IRF3 dimerization, as described previously [16]. To this end, 293T cells were transfected with a plasmid expressing an nsp1 mutant, a plasmid expressing eGFP for transfection efficiency, and a plasmid expressing STAT1α. Transfected cells were treated with IFNα for 0, 20, and 60 minutes, and extracts were analyzed by immunoblotting to 1) confirm the expression of each nsp1 mutant, and 2) to quantitate the level of phosphorylated STAT1 and the level of total STAT1. Similarly, to determine the level to which each nsp1 mutant inhibited IRF3 dimerization, 293T cells were transfected with a plasmid expressing an nsp1 mutant, a plasmid expressing eGFP for transfection efficiency, and a plasmid expressing IRF3. Transfected cells were treated with SeV for 0, 6, and 9 hours, and extracts were analyzed by immunoblotting 1) confirm the expression of each nsp1mutant, and 2) to quantitate the levels IRF3 monomers and dimers.

**Figure 6:**
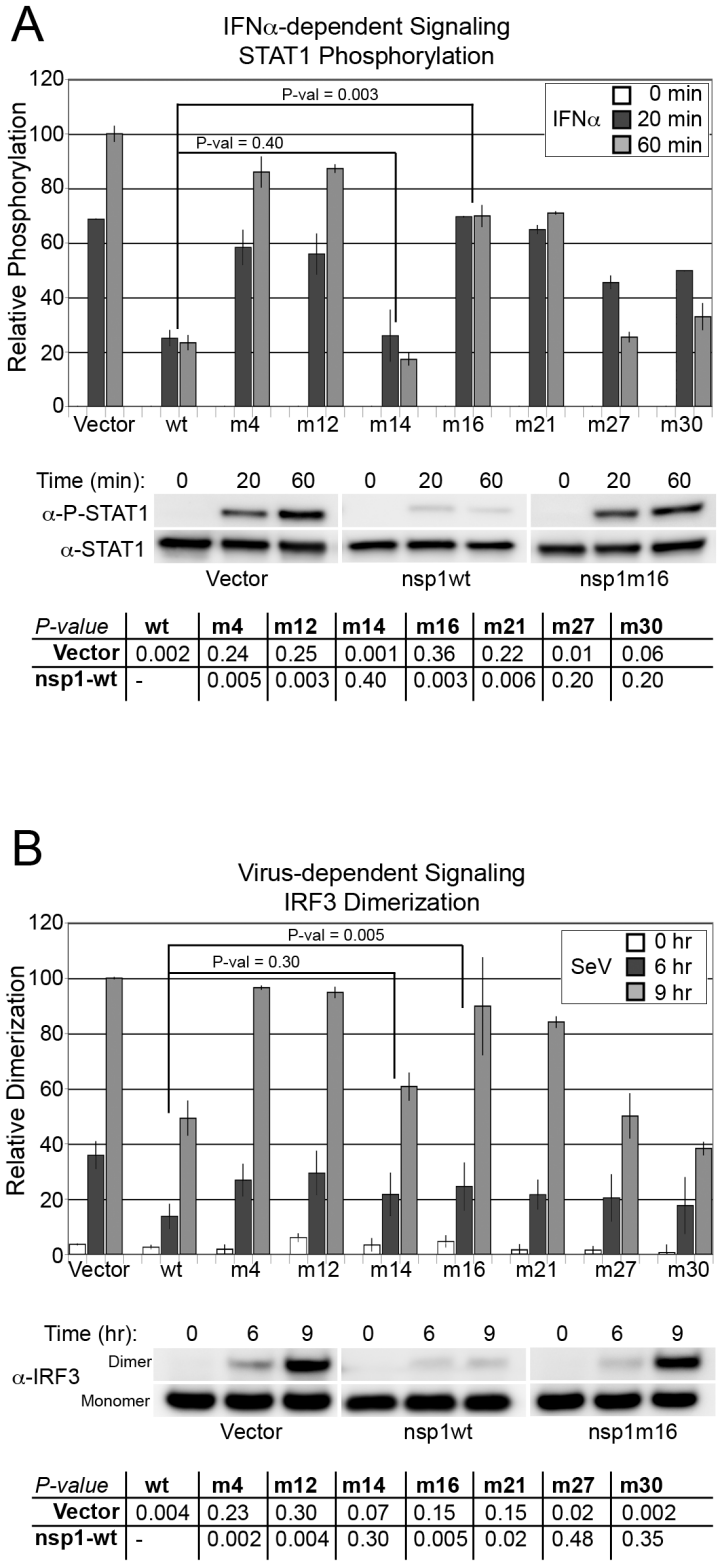
SARS-CoV nsp1 mutants show altered inhibition of signaling molecules. To confirm previous results, selected nsp1 mutants were tested for ability to directly inhibit signaling molecules. Cell extracts were separated by SDS-PAGE, and probed for levels of active phosphorylated STAT1 (P-STAT1) and for levels of total STAT1 (STAT1) (A). Cell extracts were separated by native-PAGE (B), and probed with anti-IRF3 antibody to detect both monomeric (inactive) and dimeric (active) forms. Immunoblots were quantitated and the relative activity of signaling molecules was calculated. Immunoblots confirm previous results showing that nsp1-m16 had indeed lost its ability to strongly inhibit both the phosphorylation of STAT1 and the dimerization of IRF3 after cells had been stimulated with either IFNα or SeV. Error bars are standard error; P-values are result of a t-Test and significance for all mutants is shown in (C).

As shown in Figure 6, mutants that decreased or lost their ability to inhibit antiviral signaling based on the CAT assay (Figures 3, 4, 5) indeed displayed partial or complete loss of inhibition of both STAT1 phosphorylation and IRF3 dimerization (nsp1-m4, -m12, -m21, and -m22). Expression of nsp1-m16 resulted in no decrease in either STAT1 phosphorylation or IRF3 dimerization, confirming the loss of antiviral signaling inhibition observed by CAT assay. Since nsp1-m16 retained the ability to inhibit gene expression, this mutation genetically separates the two inhibitory activities of SARS-CoV nsp1. By contrast, nsp1-m14, -m27 and -m30, which showed increased inhibition of the CAT reporter, had wildtype or partially attenuated inhibition of antiviral signaling (Fig. 4). Therefore, the increased inhibition of the CAT reporter by nsp1-m14, -m27 and -m30 must be accounted for by an inhibitory effect on CAT mRNA expression, consistent with the observed increased inhibition of β-galactosidase and luciferase activities by these mutants.

### Classification of nsp1 Mutants According to Phenotype

Table 1 is a compilation of all the nsp1 mutants generated in this study. Fold inhibition for nsp1-wt were 6.1, 1.6, 8.4 and 8.6, for β-galactosidase, luciferase, IFNα and SeV, respectively, and were set to 100 for ease of comparison. Point mutations in proteins often affect their expression levels by modulating transcription, mRNA stability, translation and/or protein stability, and we found this to be true for SARS-CoV nsp1 mutants as well. Since SARS-CoV nsp1 inhibitory activities are dose-dependent [16], we additionally normalized the measured inhibition to the levels of expression for each mutant (Table 1). Expression-normalized inhibition was used to classify the nsp1 mutants according to their ability to attenuate or increase inhibition of β-galactosidase expression and/or inhibition of antiviral signaling, leading to the identification of six broad groups, A-F, Table 1 (inhibition of luciferase expression was not considered as it was only reduced 1.6-fold by nsp1-wt). Group A mutant displayed partial attenuation of both gene expression and antiviral signaling; group B mutants had loss most inhibition of both gene expression and antiviral signaling; group C mutants; group C displayed differential effects on gene expression and signaling, with strongly reduced inhibition of signaling and maintained inhibition of gene expression; group D had increased inhibition of signaling and maintained inhibition of gene expression; group E displayed increased inhibition of both signaling and gene expression; and group F had no clear phenotype. Some mutants fell somewhat in between categories and are positioned at the edge of their groups.

The lower expression levels of some nsp1s observed in transiently transfected cells could be due to self-inhibition. Their expression could be higher during virus infection because of less self-inhibition in that context, and thus their IFN antagonism may be correspondingly higher. Consistent with the possibility of self-inhibition in transfection experiments, it was recently shown that natural SARS-CoV NP subgenomic mRNA escapes nsp1-mediated degradation, although translation of this mRNA is still inhibited by nsp1 [35]. This indicates some level of specificity for the targeting of mRNA degradation, but its molecular basis remains elusive. However, many mutants characterized in this study may be useful to elucidate the molecular basis of this function. Some mutations in SARS-CoV nsp1 changed the profile of the genes whose expression was affected: nsp1-wt strongly inhibited β-galactosidase expression but had little effect on the other genes, while other mutants showed increased luciferase inhibition with no change in β-galactosidase inhibition (e.g., nsp1-m22, -m37) and others also very strongly inhibited CAT expression (nsp1-m14, -m27, Figure 4).

## Discussion

**Figure 7:**
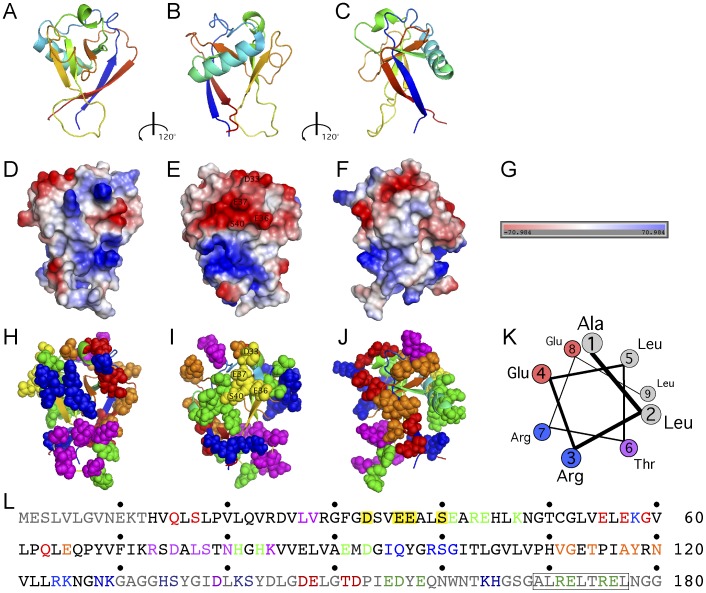
Mapping of SARS-CoV nsp1 mutants onto its 3D-structure. The structure of SARS-CoV nsp1 was solved from a.a. 13 to 127 [32] and is displayed using the PyMOL software. Top row (A–C), the backbone structure is displayed in cartoon form, it consists of a mixed parallel/antiparallel six strand β-barrel, with the α-helix (cyan) at one barrel opening and the 3_10_-helix (green) alongside the barrel. Middle row (D–F) displays protein electrostatic surface charge colored blue for positive regions and red for negative regions, with scale (G). Bottom row (H–J), atoms from amino acids that were mutated in this study are shown as space-filling spheres on top of the backbone structure in cartoon form; purple amino acids correspond to mutations that did not affect nsp1 function; blue amino acids correspond to mutations that partially attenuated both inhibition of signal transduction and inhibition of gene expression; green amino acids correspond to mutations that abolished both inhibition of signal transduction and inhibition of gene expression; yellow amino acids correspond to nsp1-m5/−m16, which maintained inhibition of gene expression but lost inhibition of IFN- and antiviral-signal transduction; orange amino acids correspond to mutations that maintained inhibition of gene expression but increased inhibition of signal transduction. Red mutations displayed increased inhibition of ISREx3CAT, β-galactosidase and luciferase reporters. The structures in each row are rotated along the vertical axis by 120°. A putative amphipathic α-helix in the C-terminus of nsp1 (a.a.169–177) is displayed in a wheel representation, a.a. are color coded: hydrophobic, grey; hydrophilic, purple; acid, red; and basic, blue (K). Primary sequence of SARS-CoV nsp1 (L): grayed amino acids correspond to regions disordered in the published NMR structure (a.a. 1–12, a.a. 128–180); amino acids were color-coded as in (H–J); the boxed sequence corresponds to the putative amphipathic α-helix displayed in (K).

SARS-CoV nsp1 could exert its multiple activities by interacting with a single factor that impinges on multiple pathways or with distinct factors that each account for some of its activities. Here, we carried out a detailed mutational analysis and identified functional surface residues of SARS-CoV nsp1 that are differentially involved in its various inhibitory functions. A summary of SARS-CoV mutations analysis is presented in Figure 7, where the mutated amino acid residues are color-coded according to their phenotypes (Table 1). None of the mutants tested displayed a significant difference in their ability to target IFN- versus virus-dependent signaling. Few mutations had no effect on nsp1 activities (purple, group F); some mutations partially (blue, group A) or almost completely (green, group B) attenuated the ability of nsp1 to inhibit both antiviral signaling and gene expression; other mutations strongly repressed the ability of nsp1 to inhibit antiviral signaling without affecting inhibition of gene expression (yellow, group C); other mutations increased the ability of nsp1 to inhibit antiviral signaling without affecting inhibition of gene expression (orange, group D); finally, some mutations (red, group E) increased both the inhibition of antiviral signaling and gene expression. Thus, our data indicate that inhibition of host gene expression and inhibition of antiviral signaling could be genetically separated and suggest that distinct but overlapping surfaces of SARS-CoV nsp1 are involved in interactions with factors that mediate inhibition of gene expression (red, green and blue) and with factors that mediate inhibition of antiviral signaling (red, orange, yellow, green and blue) (Figure 7).

Our results are consistent with SARS-CoV nsp1 targeting different host factors to mediate these inhibitory activities. One of these host factors has been identified as the 40S subunit of the ribosome and can account for nsp1 inhibition of translation [26]. However, the factors responsible for increased mRNA degradation and for inhibition of signaling remain to be discovered. In addition, a recent two-hybrid screen identified a large number of host proteins interacting with SARS-CoV nsp1, including several members of the immunophilin and calcipressin families [36]. This ability of SARS-CoV nsp1 to interact with multiple proteins despite its small size (180 residues) may be mediated in part by its disordered domains (a.a. 1–12, a.a.128–180 [32]). Intrinsically disordered domains are present in numerous proteins and their structural plasticity allows them to adopt different partial or fully ordered conformations when interacting with different ligands, increasing the size of the repertoire of proteins with which they can interact [37].

A number of viruses encode IFN antagonists that function non-specifically to target a central cellular function such as transcription or translation to prevent expression of IFNs and IFN-inducible genes. By contrast, other IFN antagonists specifically target IFN- or virus-dependent signaling, or IFN-induced proteins with antiviral function [4], [5]. In a first approximation, the mechanism by which IFN production/action are inhibited does not matter; the net result is a deficient IFN response. SARS-CoV nsp1 is unusual in that it inhibits gene expression and it inhibits both virus- and IFN-dependent signaling [16]. A mutant such as nsp1-m16, which lacks any ability to inhibit antiviral signaling while maintaining inhibition of gene expression, may prove useful to determine the relative importance of various inhibitory functions in evasion from the innate immune response and in overall virulence. Inhibition of signaling may be a more efficient strategy to antagonize the antiviral response than inhibition of gene expression due to the relative scarcity of signaling proteins vs. the abundance of components of the gene expression machinery, such as ribosomes. Thus, the levels of nsp1 necessary to inhibit a signaling target will be reached earlier during the replication cycle than those necessary to inhibit translation. The need for IFN antagonists to reach a threshold level before becoming effective is illustrated by the observation that at an early time point during SARS-CoV infection, IRF3 is observed to translocate to the nucleus whereas it is excluded from the nucleus at a later time point [38].

A specific inhibition of the IFN genes as opposed to a general inhibition of gene expression is expected to alter the immune response. Indeed, even subtle changes in IFN basal and induced expression are associated with disease states [39], and thus more selective inhibition of the induction of IFNs and IFN-inducible genes as observed with SARS-CoV may also impact the severity of the disease it causes. It has been shown that genes encoding inflammatory mediators are expressed at relatively much higher levels in SARS patients and *in vitro* tissue culture models than IFN genes when compared with other viral infections [40]–[45]. SARS-CoV nsp1 inhibitory effects on antiviral signaling [16] and its stimulatory effect on the Calcineurin/NFAT pathway [36] likely contribute to the increased production of inflammatory mediators in SARS patients. Such an exaggerated inflammatory response leading to a “cytokine storm” is thought to account at least in part for SARS-CoV morbidity and mortality [42], [43], [45], [36].

Long-term replication of a virus in a host population may result in evolution towards decreased virulence, defined as harm to the host, as less damage to the host may increase the fitness of a virus. Consistent with such an evolutionary successful solution, several human coronaviruses (HCoVs) have an established niche in humans and cause no or mild disease in healthy adults [7]–[9], [11], [12]. By contrast, SARS-CoV emergence in humans from another animal niche caused severe disease and even death in a substantial fraction of infected individuals [7]–[10]. Significantly, several SARS-CoV nsp1 mutations increased its ability to function as an IFN antagonist (e.g., nsp1-m14, -m27 and -m30, Figures 4&5). This increase was not due to increased inhibition of signaling (Figure 6), but was due to increased inhibition of gene expression (β-galactosidase, luciferase and CAT, Figures 4&5). Thus, our work indicates that a change in a few amino acids of SARS-CoV nsp1 was sufficient to substantially increase its overall IFN antagonism, which may lead to increase virulence. Comparable mutations may occasionally emerge in other human coronaviruses and lead to increased virulence, before selective pressure eventually eliminates such mutants. Other mutations tested decreased SARS-CoV nsp1 IFN antagonism and such mutations may have been part of SARS-CoV adaptation to the human population had it not been eradicated by public health measures.
